# Clinical applications and perceptions of bioceramics in endodontics: a cross-sectional survey

**DOI:** 10.2340/biid.v12.45127

**Published:** 2025-12-17

**Authors:** Kawther Belhaj Salah, Dorra Samet, Hanen Boukhris, Roua Habbachi, Imen Gnaba, Souha Ben Youssef

**Affiliations:** a Department of Restorative Dentistry and Endodontics, Faculty of Dental Medicine of Monastir, University of Monastir, LR12SP10 University Hospital of Farhat Hached -University of Sousse, Sousse, Tunisia; b Private Practice. Research Laboratory: LR 12SP10: Functional and Aesthetic Rehabilitation of Maxillary, University of Sousse, Sousse, Tunisia; c Department of Prosthodontics, Faculty of Dental Medicine, University of Monastir, LR12SP10 University Hospital of Farhat Hached -University of Sousse, Sousse, Tunisia; d Department of Oral Surgery, Faculty of Dental Medicine, University of Monastir, LR12SP10 University Hospital of Farhat Hached -University of Sousse, Sousse, Tunisia

**Keywords:** Bioceramics, calcium silicate-based sealer, clinical Applications, clinical perception, mineral trioxide aggregate

## Abstract

**Background:**

Bioceramic materials have emerged as a significant advancement in endodontics, offering excellent biocompatibility, sealing ability, and bioactivity.

Their use has expanded in recent years, particularly for pulp capping, perforation repair, and regenerative procedures.

**Objectives:**

This study aimed to assess the knowledge of bioceramics among private practice dentists, evaluate the frequency of use of sealing and filling bioceramics, and examine the applied clinical protocols as well as potential shortcomings.

**Materials and methods:**

This cross-sectional study was conducted according to Strengthening the Reporting of Observational Studies in Epidemiology (STROBE) guidelines, based on an epidemiological Knowledge, Attitudes, and Practices (KAP) survey. It was carried out among Tunisian private practice dentists. Data were processed and analyzed using Excel 2007 and Statistical Package for the Social Sciences (SPSS) Statistics 21.0. The sample size was calculated using the standard formula for estimating a proportion in a cross-sectional study, and participants were randomly selected. Participation was voluntary, anonymous, and confidential.

**Results:**

A total of 200 dentists participated. Only 54.5% reported using sealing bioceramics, with 35.05% using them in all cases, 19.94% for periapical lesions, and 16.92% for root perforations. Approximately half of the dentists (53.01%) observed faster healing when using these sealers. Regarding filling bioceramics, 67.5% of participants used them, with a preference for MTA. These materials were indicated for various clinical situations, including repair of floor perforations, pulp capping, apexification of immature permanent teeth, and repair of root perforations. However, 45.61% of participants reported treatment failures when using these bioceramics, with failure to adhere to one of the endodontic treatment steps identified as the primary cause.

**Conclusion:**

This study concluded that younger dentists tend to use bioceramics more frequently, filling bioceramics are better known than sealing ones, and most participants are familiar with the clinical indications of these materials. It is essential to provide further guidance and recommendations on the clinical application of bioceramics to optimize treatment outcomes.

## Introduction

Endodontics is constantly evolving thanks to scientific advances and the introduction of new materials. Among these, bioceramics have significantly transformed dental practice due to their exceptional properties [1].

Bioceramics refers to a significant subset of biomaterials, and includes materials that can be classified as bioinert, bioactive or biodegradable according to the interaction with surrounding tissues. Composed mainly of calcium phosphates, calcium sulfates, and bioactive glasses, bioceramics have emerged as an essential material in dentistry thanks to their remarkable physical, chemical, and biological properties. In endodontics, calcium silicate-based cements or sealers only display a small subset of bioceramics, which are mainly bioactive [1]. In addition to their mechanical and chemical performance, these cements play an essential role in endodontic treatments by promoting tissue regeneration [1].

Mineral Trioxide Aggregate (MTA), the pioneering bioceramic material in endodontics, revolutionized the field by enabling single-session apical barrier formation and optimal sealing [2, 3]. White MTA (ProRoot MTA) was later introduced to prevent tooth discoloration, and despite its widespread use and biological advantages, MTA has limitations such as prolonged setting time and difficult handling [4–8]. Biodentine, introduced in 2009, represents a newer generation of tricalcium silicate-based cements with improved handling, faster setting, and enhanced mechanical properties [4, 6].

Bioceramic sealers complement these materials by offering excellent sealing ability, biocompatibility, dimensional stability, and bioactivity [7, 8]. Their hydrophilic nature allows use in moist canals, while sustained calcium ion release enhances antimicrobial and regenerative effects [7, 9, 10]. Available in powder-liquid or premixed injectable forms, bioceramic sealers achieve hermetic obturation of complex canal systems and limit microbial colonization, showing superior activity against *Enterococcus faecalis* and other endodontic pathogens [8, 11].

Due to their excellent sealing ability and biocompatibility, these cements are employed for several clinical situations including pulp capping in primary and permanent teeth, root-end filling, perforation repair, and apical plug for teeth with open apices [1, 9].

In this context, a cross-sectional study was conducted among private dental practitioners in Tunisia to assess their level of knowledge and evaluate their attitude and clinical use of bioceramic sealers and filling materials.

To our knowledge, this study is the first to explore both the perception and clinical application of bioceramic sealers and filling materials among dental practitioners in Tunisia and across North Africa, underscoring its significance in addressing a gap in the endodontic literature.

## Materials and methods

This cross-sectional study was conducted according to STROBE guidelines [12]. This questionnaire-based survey was performed from November 2024 to January 2025. The questionnaire was distributed to private dentists either in person or via an online questionnaire. A total of 200 dentists were asked to participate in the survey. After explaining the aims and objectives of the study, verbal consent was obtained from participants before the survey began. Dentists were assured that their information would remain confidential and that the questionnaire was anonymous.

The questionnaire consisted of 21 questions divided into four sections:

- General Information (six questions): demographic and professional characteristics such as age, gender, years of practice, type of practice, and specialty.
- Knowledge (four questions): understanding of bioceramic materials, indications, and clinical properties.
- Attitudes (two questions): perceptions of bioceramics, willingness to use them, and perceived advantages or limitations.
- Practices (nine questions): actual use of bioceramic materials in daily practice, techniques, and frequency of application.

Appendix 1 provides the full questionnaire used in this study.

### Participants and selection criteria

The inclusion criteria targeted all general dentists practicing in private dental clinics. Dentists working in hospitals or university hospitals (including employed dentists, interns, and residents), as well as endodontist and other specialist were excluded. Participants were selected randomly, and participation was voluntary, anonymous, and confidential.

### Sample size calculation

The sample size was calculated using the standard formula for estimating a proportion in a cross-sectional study:


$$n=\frac{Z^{2}.p.\left(1-p\right)}{E^{2}}$$

where *Z* is the standard normal deviate at a 95% confidence level (1.96), *p* is the expected prevalence, and *E* is the desired margin of error (0.05). Based on data from a previous epidemiological study reporting a 13.3% frequency of bioceramic sealer use among general dental practitioners [13], the estimated sample size was 178.

Thus, a minimum of 178 participants was required to ensure a reliable estimation of bioceramic usage frequency with 95% confidence and a 5% margin of error.

The data collected was recorded and analyzed using SPSS version 27.0 software.

The analysis was conducted in two phases:

- **Descriptive analysis** was used to summarize the distribution of qualitative variables, expressed as frequencies and percentages.
- **Analytical tests** were applied to assess associations between categorical variables, using appropriate statistical tests.

## Results

A total of 200 questionnaires were collected, among them 57.5% had been completed by males and 42.5% by females. The sex ratio was equal to 1.3. The age distribution of the respondents was in between 24 and 65 years. About 76% of the study population belonged to the age group of 30–50 years, while 16.5% were between the ages of 24 and 30 years. However, dentists over the age of 50 represented the smallest proportion of our sample, at 7.5%.

Dentists practicing in northern Tunisia represented the majority of our sample, accounting for 50.5%, followed by those working in the coastal region, accounting for 23.5%.

When it comes to participation in postgraduate training courses, the majority of respondents (71.5%) reported not having attended any such courses related to bioceramic materials in endodontics, whereas only 28.5% had received postgraduate training in this area (Table 1).

**Table 1:** Characteristics of the respondents (n = 200).

Characteristics	*n* (%)
**Gender**	
Male	115 (57.5)
Female	85 (42.5)
**Age (years)**	
24–30	33 (16.5)
31–50	152 (76.0)
> 50	15 (7.5)
**Practice region**	
Northern Tunisia	101 (50.5)
Coastal region	47 (23.5)
Other regions	52 (26.0)
**Postgraduate training in bioceramics**	
No	143 (71.5)
Yes	57 (28.5)

### Use of bioceramic sealers in endodontics

Among the participating dental professionals, 54.5% reported using bioceramic sealers for root canal obturation, while 45.5% did not. The majority of those who used them (76.1%) preferred the injectable form, followed by 18.5% who chose the powder-liquid form, and 5.3% who employed both formats.

**Figure 1:**
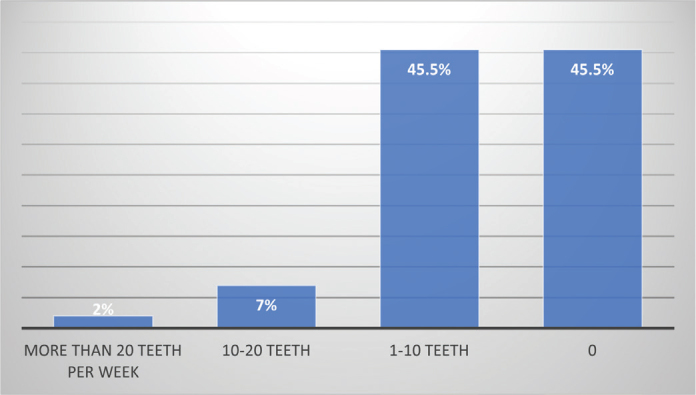
Dentist distribution based on the frequency of use of bioceramic-based endodontic sealers in root canal fillings.

Figure 1 shows that the use of bioceramic sealers varied among clinicians, with a substantial proportion reporting little to no use on a weekly basis.

**Figure 2:**
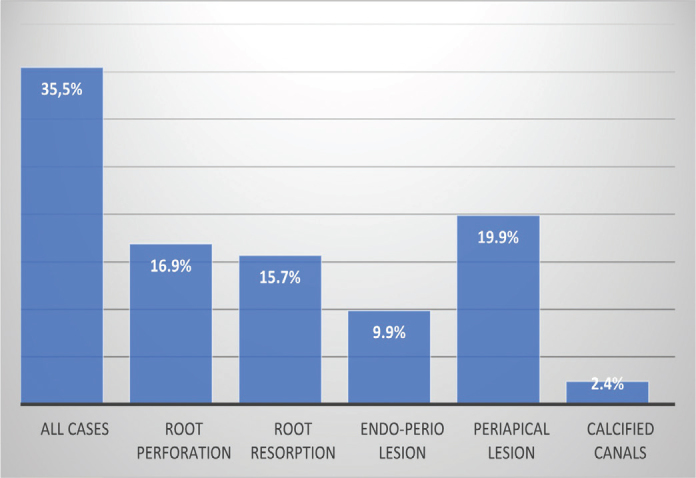
Indications for bioceramic-based endodontic sealers according to the survey participants.

As shown in Figure 2, while some clinicians use bioceramic sealers for all endodontic cases, others prefer them for specific situations such as periapical lesions or root perforations.

As for perceived clinical outcomes, 53% of respondents reported faster healing when using bioceramic sealers, while 12.6% observed no significant difference compared to conventional sealers.

### Use of bioceramic filling in endodontics

Among the respondents, 67.5% reported using bioceramic materials for root repair procedures, while 32.5% did not incorporate them into their daily clinical practice. When specifying the type of bioceramic material used, 63.5% of practitioners reported using MTA, 17.52% used Biodentine, and 18.98% used both of them.

With regard to how long these materials had been in use, 50.3% of the dentists had been using them for 2 years, 26.6% for 1–2 years, and 23% had adopted them only recently. Regarding clinical frequency, 36.5% of practitioners stated they had never used bioceramic repair materials, 61.5% used them in 1 to 5 cases per week, and only 1% applied them in 5 to 10 cases weekly.

**Figure 3:**
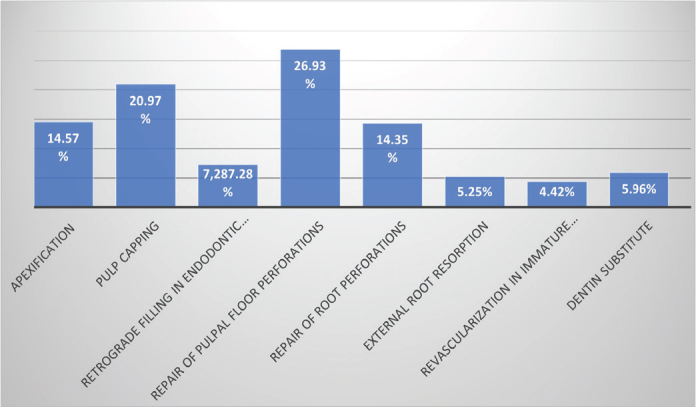
Clinical indications for the use of bioceramic filling material reported by practitioners.

As illustrated in Figure 3, the primary clinical applications of bioceramic materials included repair of furcation or pulpal floor perforations and pulp capping, while apexification and root perforation repair were reported less frequently.

**Figure 4:**
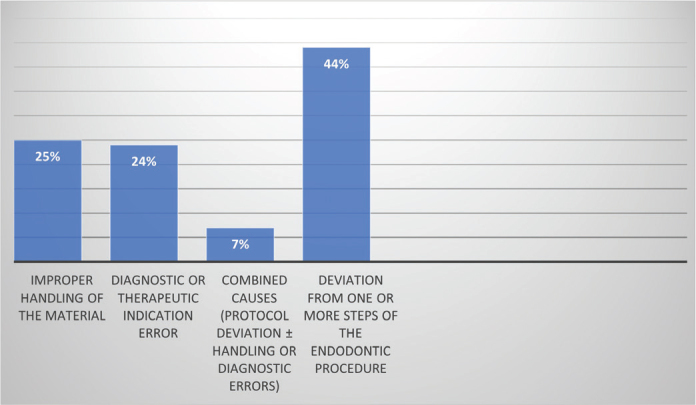
Participants’ perspectives on causes of treatment failure.

Regarding perceived outcomes, most practitioners reported no treatment failures with bioceramic materials. Among those who had encountered failures, the primary contributing factors were deviations from endodontic protocols, incorrect handling of the materials, and diagnostic or therapeutic errors, as shown in Figure 4.

In order to further investigate the data, an analytical study was conducted by statistical tests and cross-referencing the data to explore potential associations between variables.

In fact, the use of bioceramic sealers was comparable between practitioners with less than 10 years of experience (54.5%) and those with more than 10 years (54.4%), with no statistically significant difference between the two groups.

In contrast, the use of bioceramic filling materials varied more distinctly with experience. A higher proportion of practitioners with less than 10 years of practice (72.7%) reported using these materials, compared to 59.5% among those with more than 10 years in practice. Fisher’s test revealed a statistically significant association, indicating that more younger practitioners had adopted bioceramic repair materials in their clinical practice (Table 2).

**Table 2:** Statistical tests assessing the relationship between the use of bioceramic repair materials and years of clinical experience.

Test	Value	df	Asymptotic significance (2-sided)	Exact significance (2-sided)	Exact significance (1-sided)
Pearson chi-square	3.816^a^	1	0.051		
Continuity correction^b^	3.236	1	0.072		
Likelihood ratio	3.780	1	0.052		
Fisher’s exact test				0.064	0.036
Number of valid observations	200				

Notes:

a 0 cells (0.0%) have expected count less than 5.

b Computed only for a 2 × 2 table.

Regarding geographic location, the use of bioceramic sealers ranged from 52.2% in the North and South of Tunisia to 52.9% in the Center, and 61.7% in the coastal region. However, Pearson’s chi-square test showed that this variation was not statistically significant (Table 3).

**Table 3:** Statistical test showing the relationship between the use of bioceramic sealer and the practice region.

Test	Value	df	Asymptotic significance (2-sided)
Pearson chi-square	1.288^a^	3	0.732
Likelihood ratio	1.300	3	0.729
Number of valid cases	200		

a: Statistically significant difference

Similarly, the use of bioceramic repair materials varied by region: 62.8% in the North, 70.6% in the Center and 69.6% in the coastal region. Despite these differences, Fisher’s test did not show a significant association between geographic location and the use of bioceramic repair materials (Table 4).

**Table 4:** Statistical test showing the relationship between the use of filling bioceramics and the practice region.

Test	Value	df	Asymptotic significance (2-sided)
Pearson chi-square	3.014^a^	3	0.390
Likelihood ratio	3.096	3	0.377
Number of valid cases	200		

## Discussion

This cross-sectional study is the first research conducted among private practice dentists in Tunisia and across North Africa to assess their knowledge, attitude and practice when using bioceramic sealers and filling materials, underscoring its significance in addressing a gap in the endodontic literature. The results revealed that the use of bioceramics remains limited, with a notable preference for MTA despite the recent advancements offered by second-generation bioceramics. This survey also highlighted the main factors contributing to endodontic treatment failure when using bioceramics, with the most significant being the failure to adhere to one or more essential steps of the endodontic procedure. Only two other studies have explored related topics: one assessed the use of calcium silicate-based sealers in non-surgical endodontic treatment [14], while the other evaluated the use of MTA and Biodentine [15]. To our knowledge, no recent study has investigated both knowledge, attitude, practice and procedural preferences simultaneously, which underscores the relevance of our findings.

The analysis of the general profile of the participating dentists revealed a noticeable gender imbalance, with a predominance of female practitioners. Additionally, the majority of respondents belonged to the middle-aged group, suggesting that the survey primarily captured the perspectives of practitioners with a certain level of professional experience, likely reflecting those actively engaged in clinical endodontics.

Regarding the use of bioceramics in daily practice, an international survey was conducted among general dentists and endodontists. It revealed that 41% of general dentists reported using these materials [14]. In contrast, our study revealed that 54.5% of practitioners used bioceramic sealers, while 67.5% used bioceramic filling materials. Notably, the use of bioceramics was more common among dentists with less than 10 years of clinical experience.

Our study also investigated the types of bioceramic materials used. Comparable surveys in other countries have reported similar tendencies. For instance, Ha et al. [15] conducted a survey among Australian endodontists and found that MTA was the most commonly used material, while Biodentine was less frequently adopted due to limited familiarity and handling challenges. These findings are consistent with our results, where MTA remains the preferred material among practitioners, reflecting a global pattern of slow integration of second-generation bioceramics despite their improved properties.

On the other hand, this study further explored the use of bioceramic sealers, highlighting their growing relevance in endodontic practice. In fact, 76.1% of respondents reported using the injectable form of bioceramic sealers, 18.5% used the powder-liquid form, and 5.3% reported using both. This more common use of the injectable form could be explained by the superior handling properties and reliable sealing ability of the injectable form compared to the powder-liquid form, which is more technique-sensitive and operator-dependent. In fact, when using powder-liquid forms, such as BioRoot RCS (Septodont), including a mixing solution, strict adherence to the manufacturer-recommended powder- to-liquid ratio is essential, as deviations may result in premature or delayed setting [11]. Studies by Camilleri et al. have shown that the final porosity of the set material is influenced not only by this ratio but also by the mixing technique employed [6]. In this context, 25% of the dentists declare that improper handling of the material is the main reason of failure in their endodontic treatments.

More recently, the use of bioceramic sealers in endodontics has been increasingly indicated for a variety of clinical situations. According to this study, the primary use of these sealers was in the management of periapical lesions. This finding clearly indicates that most practitioners are aware of the properties of bioceramic sealers, which have been shown to be better than those of conventional root canal sealers [9, 16]. In fact, in cases of large periapical lesions, studies reported favorable outcomes in patients treated with bioceramic sealers and a superior periapical healing, with a significant reduction in lesion size [17, 18].

Furthermore, bioceramic sealers have also proven effective in cases involving root resorptions. Their high mechanical strength contributes to the structural reinforcement of affected teeth and calcium ion release promotes the repair of dental tissues while raising the local pH, favoring remineralization and limiting bacterial proliferation [18, 19]. According to our findings, 15.71% of practitioners employ bioceramic sealers for the management of root resorptions. Bioceramic sealers are also indicated in cases of endo-periodontal lesions and the results of this study show that participants were quite aware of this indication.

A recent Bulgarian study conducted by Stefanova et al. [21] evaluated the clinical use of bioceramic sealers. Although 95% of participants reported being familiar with bioceramic materials, only 51% incorporated them into their clinical practice. This highlights a clear gap between theoretical knowledge and clinical application. These findings align with our study, in which bioceramic use remained limited despite a fair level of awareness, especially among private practitioners. This underscores the pressing need for targeted training programs and practical guidelines to support the integration of bioceramics into routine endodontic procedures.

With regard to the bioceramic filling materials, the respondents of the survey found the primary indication for its use in endodontics to be the repair of pulpal floor perforation. In fact, pulpal floor perforation represent pathological communications between the root canal system and the periodontal tissues that significantly compromise the prognosis of the affected tooth. Therefore, prompt and effective sealing of the perforation is essential to ensure a favorable outcome [22, 23]. Both MTA and Biodentine are effective for repairing pulp chamber floor perforations [24, 25]. In this context, 26.9% of surveyed dental practitioners reported the use of bioceramic materials for the management of pulp chamber floor perforations. Consistently, previous studies have shown that bioceramic filling materials remains the predominant material for perforation repair, reflecting its continued preference among clinicians due to its proven sealing ability and biocompatibility [15]. According to earlier studies, 87.8% of general dentists preferred MTA for perforation repair, followed by 6.1% who opted for Biodentine [15]. These findings closely align with our results, where 63.5% of practitioners reported using MTA, 17.5% used Biodentine.

The survey results indicates that also bioceramic repair materials are indicated for the management of root perforation. MTA remains the material of choice in such cases, as well as for perforations in the middle or apical third. Similar observations have been reported in the scientific literature [18].

In addition to managing iatrogenic perforations, this study revealed that bioceramic filling materials were commonly employed by dentists for root-end fillings and apical barrier formation in immature permanent teeth. These findings are consistent with the indications reported in similar studies [15]. Although Biodentine provides a quicker setting time and better esthetic outcomes, especially in anterior teeth [26, 27] MTA was the most frequently used in these cases Ha et al. [15] reported similar results as observed in our study. According to the authors only 45.5% of practitioners performed root-end fillings, with MTA being the most frequently used material (78.3%).

Finally, according to our survey, 45.6% of respondents reported no failures when using bioceramic materials, while 18.7% experienced failures and 35.6% were neutral. The most frequently cited cause of failure was the inadequate execution of one or more steps of the endodontic procedure, followed by improper handling of the material and diagnostic or therapeutic misjudgment. A small number of practitioners reported multiple contributing factors.

In fact, several studies confirm that many factors can influence the clinical success of bioceramic-based endodontic treatments. These include the quality of canal cleaning, shaping, as well as the irrigation protocols and the integrity of the final restoration [28, 29]. Proper manipulation of the material, particularly adherence to the recommended powder-to-liquid ratio and working time, is also critical [30]. In addition, accurate diagnosis and appropriate case selection, such as evaluating the location and size of a perforation, are essential for favorable outcomes [25].

## Conclusion

This study shows that the use of bioceramics among private dentists in Tunisia remains limited, particularly regarding bioceramic sealers, with a preference for MTA over newer materials such as Biodentine. Treatment failures were primarily associated with deviations from recommended protocols or improper handling rather than inherent material limitations, highlighting a gap between theoretical knowledge and clinical practice. These findings underscore the need for clearer clinical guidelines and expanded postgraduate education and hands-on training to promote the effective and evidence-based use of bioceramic materials in everyday endodontic practice.

## Strengths and limitations of the study

One of the main strengths of this study is its ability to capture current clinical practices and preferences concerning the use of bioceramic materials in endodontics, based on direct feedback from practitioners. It provides an informative overview of how these materials are used in daily practice across a range of clinical situations. Nevertheless, some limitations should be acknowledged. As the data are self-reported, they may be influenced by response bias, and the absence of clinical outcome verification limits the ability to assess the accuracy of the reported practices. In addition, the sample may not fully represent the diversity of endodontic practice, particularly among specialists or practitioners working in different clinical settings or in different geographical settings.

## Implications for future research

The findings of this study point to the need for further research into the clinical performance of bioceramic materials in different endodontic applications. Long-term, prospective studies are required to evaluate their success and durability, especially in demanding procedures such as regenerative endodontics. Future research should also investigate the factors that influence the choice of material by clinicians, including professional training, material availability, and perceived reliability. Comparative clinical trials involving MTA, Biodentine, and newer bioceramic materials would help to refine clinical recommendations and strengthen the evidence base guiding endodontic treatment decisions.

## Key messages

- This study provides the first data on the perception and clinical use of bioceramic sealers and filling materials among dentist in Tunisia and North African countries.
- The findings highlight a growing interest in bioceramic materials, yet reveal variations in their adoption depending on practitioners’ experience, training, and clinical setting.
- Increased educational efforts and evidence-based guidelines could support the optimal integration of bioceramic materials into endodontic practice in the region.

## Supplementary Material


