# Supplementary material for: Clinical applications and perceptions of bioceramics in endodontics: a cross-sectional survey
Source: Biomater Investig Dent. 2025 Dec 17;12:45127. doi: 10.2340/biid.v12.45127 (PMC12729095; doi:10.2340/biid.v12.45127)
Supplement: Supplementary file 1 [file BIiD-12-45127-s1.pdf]

Supplementary material has been published as submitted. It has not been copyedited or typeset by Biomaterial Investigations in Dentistry.

KAP Questionnaire on Bioceramic Use in Endodontics

I. General Information

1. Gender

a) Male b) Female

2. Age group

a) 24–30 b) 30–50 c) Over 50

3. Years of clinical experience

a) 0–5 b) 6–10 c) 11–15 d) More than 15

4. Practice location

a) North-East (Tunis, Nabeul, Hammamet)

b) North-West (Bizerte, Beja, Jendouba, Kef)

c) Central region

d) Coastal region

e) South

5. Have you received any postgraduate training on bioceramic materials in endodontics?

a) Yes b) No

6. Would you be interested in future training on the use of bioceramics in endodontics?

a) Yes b) No

II. Knowledge

1. Do you use bioceramic-based endodontic sealers for root canal obturation?

a) Yes b) No

2. If yes, what formulation do you use?

a) Powder-liquid b) Injectable

3. What indications are you aware of for bioceramic sealers? (Multiple choice)

a) All cases

b) Periapical lesions

c) Root resorptions

d) Calcified canals

e) Endo-perio lesions

f) Root perforations

4. In your opinion, how do bioceramic sealers influence endodontic healing?

a) Faster healing compared to other sealers

b) No difference in healing

c) I do not follow up healing outcomes

III. Attitudes

1. Do you consider bioceramic materials effective in improving endodontic outcomes?

a) Yes b) No c) Not sure

2. Do you trust second-generation bioceramics like Biodentine over traditional materials (e.g. MTA)?

a) Yes b) No c) No opinion

IV. Practices

1. How often do you perform endodontic treatments per week?

a) 1–10 teeth b) 11–20 teeth c) More than 20 teeth

2. How often do you use bioceramic sealers in your root canal obturations?

a) Never b) 1–10 teeth/week c) 11–20 teeth/week d) >20 teeth/week

3. Do you use bioceramic filling materials (e.g. MTA, Biodentine)?

a) Yes b) No

4. If yes, which material do you use?

a) MTA b) Biodentine c) Both

5. How long have you been using bioceramic filling materials?

a) Less than 1 year b) 1–2 years c) More than 2 years

6. How often do you use bioceramic filling materials?

a) Never b) 1–5 teeth/week c) 6–10 teeth/week d) >10 teeth/week

7. In which clinical situations do you use bioceramic filling materials? (Multiple choice)

a) Direct or indirect pulp capping

b) Dentin substitute

c) Floor perforation repair

d) Root perforation repair

e) External root resorption

f) Apical plug in apexification

g) Retrograde filling in apical surgery

h) Revascularization

8. How do you evaluate the outcomes of cases treated with bioceramic filling materials?

a) No failures observed

b) I have encountered failures

c) Neutral opinion

9. What do you believe is the most common cause of treatment failure using bioceramic materials?

a) Improper endodontic protocol

b) Handling errors

c) Incorrect diagnosis or indication
